# The Portrayal of Periocular Aging Changes in Renaissance Art: A Case–Control Study to Compare Northern vs Southern Schools of Art

**DOI:** 10.1093/asjof/ojag181

**Published:** 2026-09-18

**Authors:** Oshin Bansal, Marie-Anne Mancio, Laxmi Raja, Mia Z H Maierovits, Arnaud Potvin, Daniel G Ezra

## Abstract

The 15th and 16th centuries in Europe were marked by a resurgence of focus on the arts and humanities and hearkened back to ancient cultures with new allusions and influences. This period was called the Renaissance, and although the Renaissance is often thought of as an Italian phenomenon, the entirety of Europe experienced a parallel shift marked by varying characteristics. In comparison, Italy's and Northern Europe's Renaissances differ in their approach to the newly burgeoning sense of representation in the art world. Although the Italian Renaissance focused on idealized subject matter and subjects that veered away from classic religious concepts, the Northern Renaissance explored altarpieces and realistic daily portraits of everyday life. The aim of this study was to ascertain which among Northern and European Schools of Renaissance Art had a more realistic depiction of facial aging by employing scientific grading systems to objectively measure various periocular signs of aging. An age-matched case–control study was conducted at a tertiary eye care institute in collaboration with an art historian. Twenty paintings of each school of art were studied and graded for dermatochalasis and tear trough–eyelid bag complex, and 2 schools were compared. Dermatochalasis (*P* < .001) and Bulging scores (*P* = .039) showed higher values and therefore more realistic aging changes in the Northern European renaissance paintings. The Italian school of art had a more idealized and less realistic approach compared with Northern European school during the renaissance period which is realized by the scientific and more anatomically accurate periocular aging changes noted in Northern Europe art.

The relationship between beauty and aging has long been a subject of inquiry across disciplines, including philosophy, art, and medicine. Central to these discussions is the question of objectivity in the representation of beauty—how it is constructed, perceived, and measured. Although contemporary discourse frequently engages with this issue in the context of digital media, cosmetic interventions, and aesthetic medicine, the divergence between idealized portrayals of beauty and biological aging is not a modern phenomenon. The Renaissance period, in particular, offers a compelling framework for examining this dynamic, because it witnessed distinct regional approaches to the depiction of the human form, shaped by divergent cultural and philosophical underpinnings.

The Renaissance, a period marked by a revival of art, culture, and intellectual pursuits, witnessed a profound shift in the portrayal of beauty and aging. Because art serves as a mirror of its contemporary era, delving into Renaissance art and its various forms of expression becomes a valuable means to attain a more comprehensive insight into the cultural milieu that has significantly influenced many of the prevailing societal attitudes today.

Spawning out of the city-state of Florence in 14th century Italy, the Renaissance movement swiftly expanded to neighboring city-states in Southern Europe and later spanned Western and Northern Europe.^1^ Although the unification of Italy was not heralded until the 19th century, the predating city-states had considerable artistic overlap and defining characteristics in their portraiture styles that were visibly different to those of Northern Europe.^2^ Notably, Italian and Northern European artists have been historically distinguished by differing aesthetic priorities—Italian schools often idealized the human form, whereas Northern artists were known for their detailed, realistic depictions.^2,3^

To objectively ascertain the same, we embarked on a quest to map the portrayal of aging and explore the beauty standards of the Renaissance era, particularly with regard to periocular aging. Among the various facial features depicted in Renaissance portraiture, the periocular region holds particular significance because of its role in conveying signs of aging, vitality, and emotional expression. Firstly, we hypothesize that there exists a measurable divergence in the depiction of aging between the idealized representations characteristic of the Italian schools and the more realist portrayals associated with Northern European traditions. Secondly, we sought to explore the representation of women in Renaissance artwork. Although art historians have deliberated on these disciplines previously, the arguments are constrained by the subjective nature of interpretations. For instance, Bellini's celebrated portrait of the 65-year-old Doge Loredan is a realistic illustration for 1 writer; on the other hand, it possesses “all the trappings of an icon” for another author (Figure 1A).^4^ With exposure to bias and individual perspectives, it may be challenging to draw firm conclusions.

**Figure 1.**
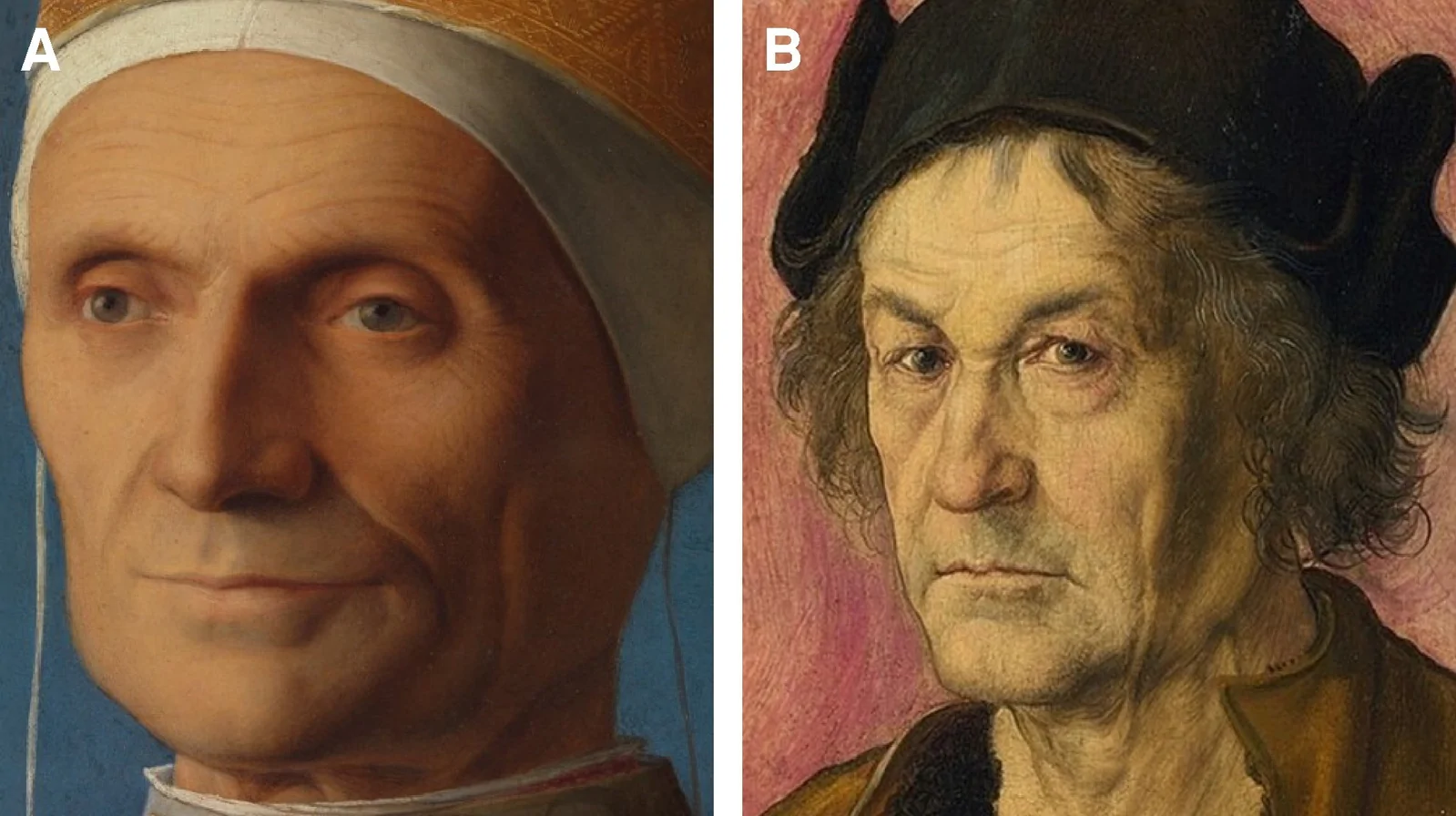
Representative images of 2 age- and gender-matched (male) portraits from (A) Italian and (B) Northern School of Renaissance Art. (A) Representative image of Bellini, G., Detail, 1501-2 Portrait of Doge Leonardo Loredan (66 years old) [oil on canvas] © The National Gallery, London. (B) Dürer, A., Detail, 1490 Albrecht Dürer the Elder (70 years old) [oil on canvas] © The National Gallery, London.

The aim of this paper is to address this limitation of subjectivity. By employing scientific grading systems, we intend to objectively measure various signs of aging in Renaissance portraits. By focusing on periocular aging, a prominent aspect of facial aging, we aim to discern patterns and unveil contours that may have been overlooked in subjective analyses. In understanding how aging was portrayed during the Renaissance, we hope to glean insights into the representation of beauty and draw inferences to societal values and norms that continue to shape modern perceptions of aesthetics. With this paper, we also intend to deliberate on the idea of regional differences in perception and standards of beauty and thus impress upon our fellow Facial Aesthetic surgeons about the individualistic approach rather than a textbook concept of beauty to inculcate in our practice.

## METHODS

This is an age-matched case–control study, conducted at a tertiary eye care hospital in London, in collaboration with an art historian. Under the expert guidance of an art historian, 20 age-matched portraits from Renaissance art schools of Northern European and Italian descent each were collected from various art collections, including Uffizi, Florence; Prado, Madrid; National Portrait Gallery, London; etc. Portraits where the exact age of sitters was unknown were excluded from the study.

Each portrait underwent assessment for the following periocular aging changes:

1. Central dermatochalasis (central hooding)
2. Lateral dermatochalasis (lateral hooding)
3. Atrophic changes around lower eyelid
4. Lower eyelid bags
5. Lower eyelid laxity

All portraits were graded according to the following published classification systems: the Jacob's eyelids classification (JEC) was used to score central dermatochalasis, whereas the lateral dermatochalasis classification (LDC) was used to grade lateral dermatochalasis.^5^ The atrophy, bulging, and laxity (ABL) classification system was employed to measure tear trough–eyelid bag complex (Supplemental Figure 1).^6^

For central dermatochalasis, the distance of the upper eyelid skin from the eyelashes was measured using the JEC grading system (the shorter the distance, the greater the degree of central dermatochalasis). The lateral dermatochalasis score was calculated using the LDC system: the relationship between the level of the lower edge of lateral dermatochalasis with respect to the line of intersection between the caruncle and the edge of the upper eyelid. For lower eyelid senile changes, the ABL classification was used which evaluated atrophic changes in lower eyelids based on the presence of a tear trough, the presence of a palpebro-malar groove, and anterior malar cheek volume loss. Lower eyelid bags were classified based on the presence of fat bags and/or malar festoons. Lower eyelid laxity was classified as laxity affecting solely the eyelid, exclusively the cheeks, or both regions.

All the portraits were individually graded by 2 masked observers, a senior oculoplastics fellow and an experienced oculoplastics and aesthetics consultant, to minimize observation bias. The scores of the aforementioned categories were compared between the northern and southern schools using the Wilcoxon signed rank test with an *α*-value of .05 to evaluate the portrayal of aging in order to shed light on wider societal and cultural perspectives. Inter-rater reliability was assessed using Cohen's *κ*.

## RESULTS

A total of 75 portraits were identified from 20 different art collections. Portraits in which the sitters’ age was unknown were excluded. Following this, a total of 40 portraits were analyzed: 20 pertaining to each school of art. Of the 20 portraits in each school, 15 constituted male sitters (75%), whereas 5 accounted for female sitters (25%). The racial composition of sitters depicted in portraits from Southern Italian schools exclusively comprised individuals identified as Caucasian (*n* = 20, 100%; Figure 2). This trend was also observed in portraits from Northern European schools (*n* = 20, 100%). The median age of muses in Southern Italian paintings was 47.5 (interquartile range [IQR] 23). In the Northern European paintings, this figure was slightly elevated at 55.0 (IQR 9.5; Table 1).

**Figure 2.**
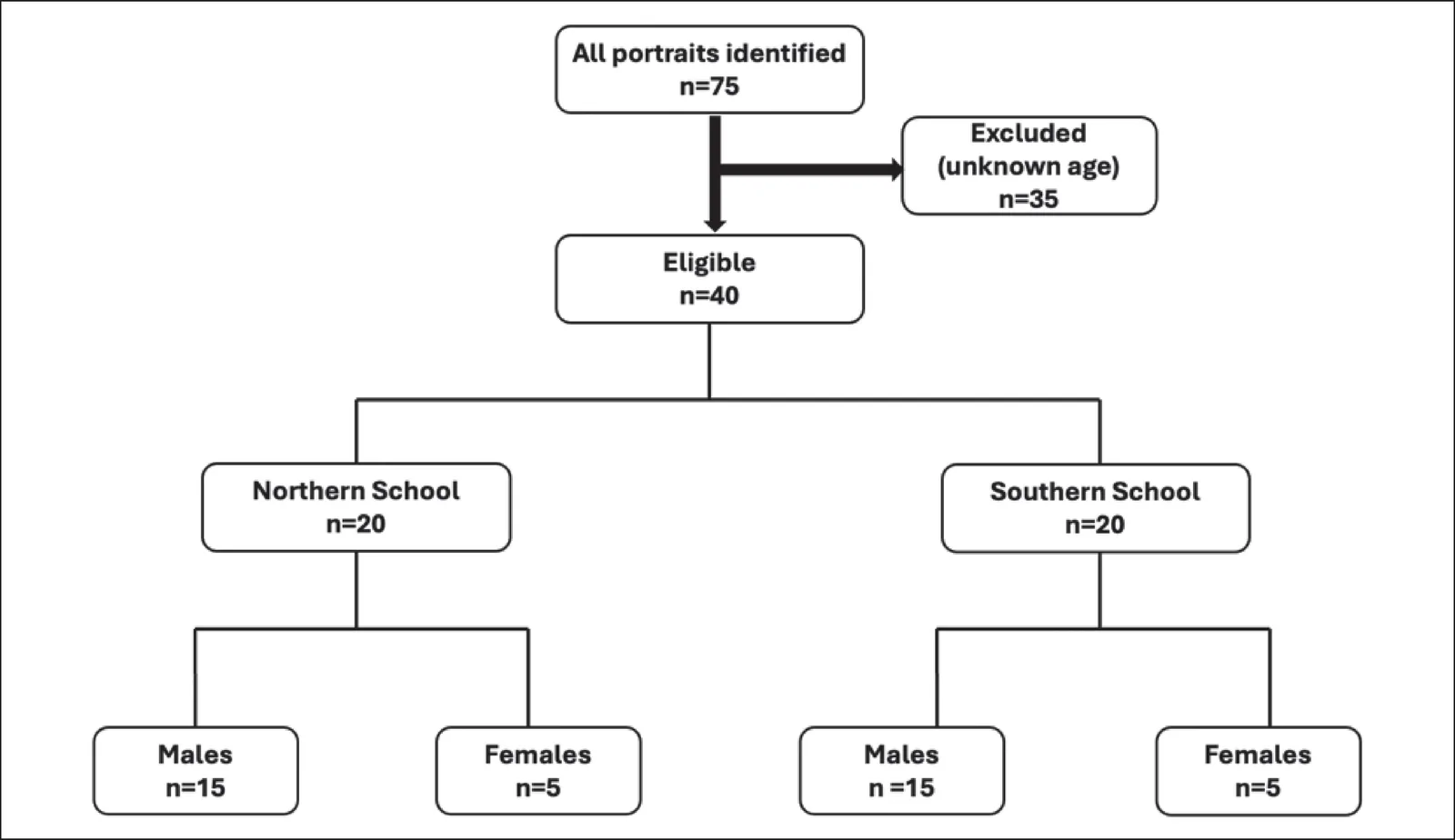
Flow diagram illustrating the eligibility/inclusion criteria and subclassification of portraits.

**Table 1.** Demographics of Portraits

Parameters	Northern schools (*n* = 20)	Southern schools (*n* = 20)
Age, years, median (IQR)	55.0 (9.5)	47.5 (23)
Gender, male, *n* (%)	15 (75)	15 (75)
Gender, female, *n* (%)	5 (25)	5 (25)
Ethnicity, *n* (%) Caucasian, White	20 (100)	20 (100)

IQR, interquartile range.

The scores for central dermatochalasis (Italian mean score—1.05 vs Northern mean score—1.55; *P* = .042), lateral dermatochalasis (Italian mean score—0.6 vs Northern mean score—2.1; *P* < .001), and lower eyelid bags (Italian mean score—1.1 vs Northern mean score—1.7; *P* = .039) were significantly lower in the Southern Italian school of portraits in comparison to the Northern European portraits. There was no statistical difference in lower eyelid atrophic scores (Italian mean score—2.15 vs Northern mean score—2.5; *P* = .24) and laxity scores (Italian mean score—1.5 vs Northern mean score—1.05, *P* = .31) between the 2 schools (Table 2, Figure 3).

**Figure 3.**
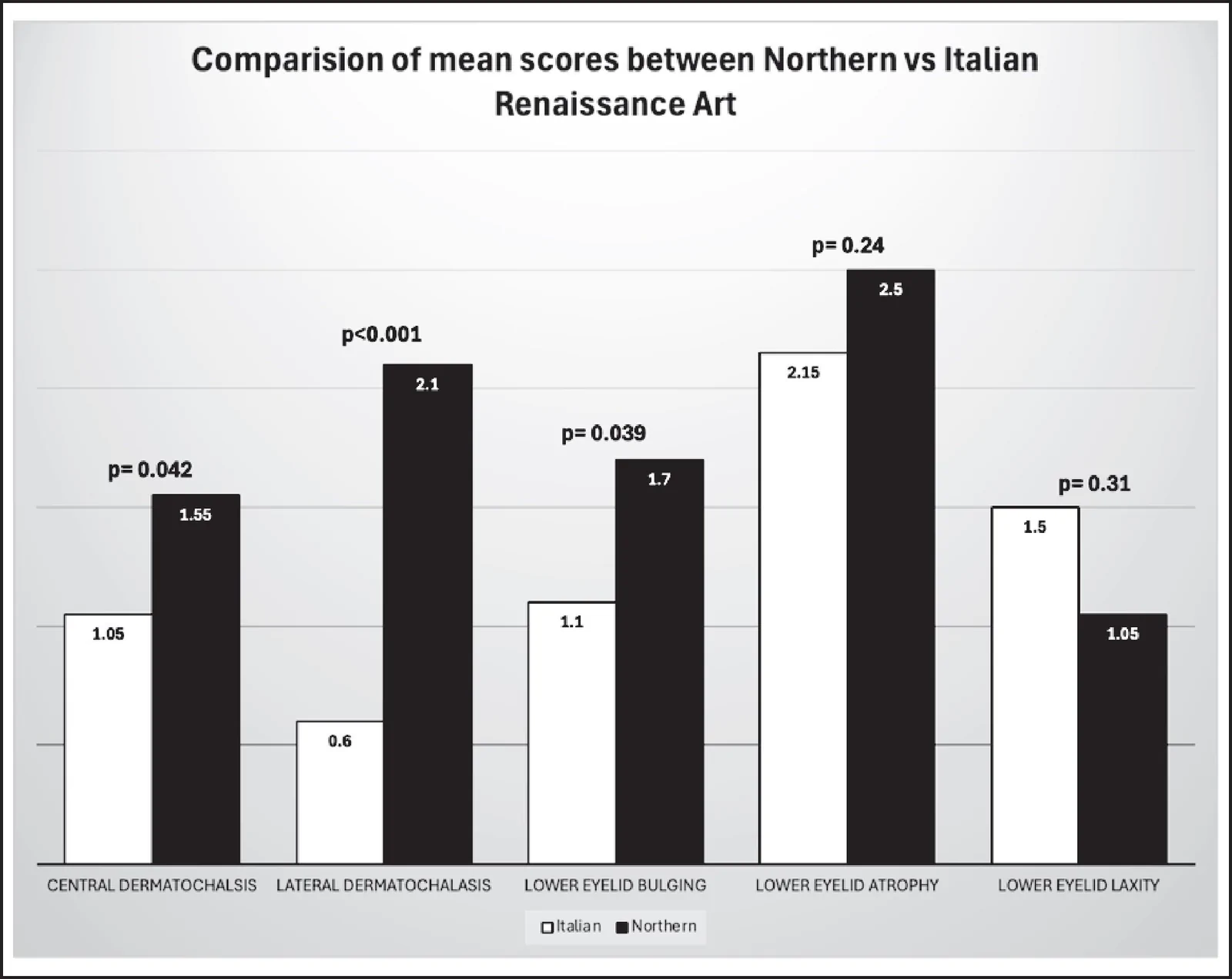
Comparison of mean eyelid assessment scores between Italian and Northern Renaissance Schools of Art.

**Table 2.** *P*-values of Various Periocular Aging Scores Compared Between Northern and Southern Schools

Parameters	*P*-values
LDC score (lateral dermatochalasis)	<.001 (3.072E-5)
JEC score (central dermatochalasis)	.042
Lower eyelid atrophy	.24
Lower eyelid bulging	.039
Lower eyelid laxity	.31

JEC, Jacob's eyelids classification; LDC, lateral dermatochalasis classification.

Inter-rater reliability was substantial for lateral dermatochalasis (*κ* = 0.69) and lower eyelid bags (*κ* = 0.73), moderate for central dermatochalasis (*κ* = 0.59) and atrophic changes (*κ* = 0.57), and slight for laxity (*κ* = 0.16).

Figure 1A and B showing representative images of 2 time-, age-, and skill-matched male portraits by 2 of the “top” artists one from each Northern and Italian School of Renaissance Art and Figure 4A and B showing representative images of 2 time-, age-, and skill-matched female portraits one from each Northern and Italian School of Renaissance Art.

**Figure 4.**
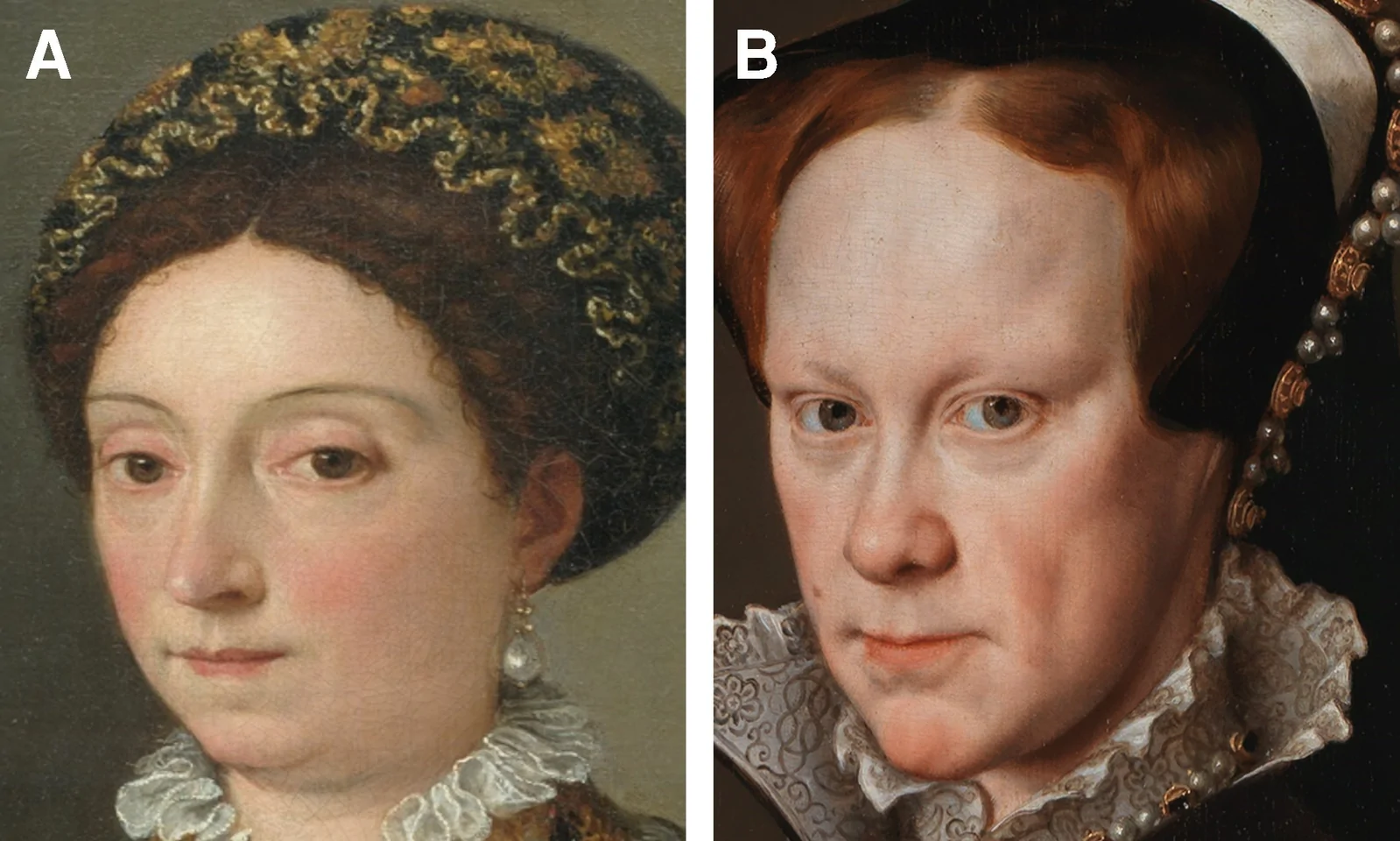
Representative images of 2 age- and gender-matched (female) portraits from (A) Italian and (B) Northern School of Renaissance Art. (A) Titian, *Portrait of Eleonora Gonzaga, Duchess of Urbino,* 44 years old, 1536-37, © Gabinetto Fotografico delle Gallerie degli Uffizi, by courtesy of the Italian Ministry of Culture, Uffizi Galleries. (B) Anthonis Mor, *Mary Tudor, Queen of England*, 42 years old, 1554, oil on panel, 109 × 84 cm, catalog number P002108, Madrid, Museo Nacional del Prado.

## DISCUSSION

The widely recognized comparisons between Southern Italian and Northern European schools of art have traditionally relied on subjective interpretations, thus posing challenges in deriving robust conclusions. This study provides a novel objective foundation to support previously observed variations within Renaissance art, using modern medical grading scales. This is the first report of Renaissance portraits undergoing objective evaluation. Through this scientific exploration, we aspire to not only deepen our understanding of the past but also to contribute to the ongoing discourse on aging and beauty, fostering a more comprehensive appreciation of the aesthetics that transcend time.

A total of 40 age- and gender-matched Renaissance portraits were evaluated in terms of periocular changes with an even split of 20 portraits from each school of art. The median age of sitters in Italian paintings was 47.5 years, and 55.0 years for Northern European sitters. It is important to highlight the comparatively younger median age of subjects in Italian portraits juxtaposed to those in Northern European portraits. This might align with the concept of Italians idealizing youth, thereby perceiving individuals as “old” at a younger age than their counterparts. The notion of “old age” starting at 50 years in the Renaissance epoch is supported by Venetian Renaissance census records which revealed a tripartite classification system for age: “putti” until age 18, “huomini” to 50 years old, and “vecchi” for age 50 and older. Interestingly, this system was exclusive to males. For females, it was simply a dual classification system marked by watersheds at ages 12 and 40. Twelve was adulthood and marriageability; 40 was considered “old age.”^7^ This system not only denotes the age in which an individual is considered elderly but also exposes the disparity in portrayal of age between men and women.

The notion that aging was generally considered undesirable in Southern Italian schools was especially prominent in the context of females. Although this is beyond the scope of the hypothesis of this paper because of the limited sample size and even smaller ratio of female portraits that were included in the study, it is rather an interesting aspect to discuss. The disparate outlook toward aging in men vs women is evident in established literature on Venetian renaissance, where older age was depicted with strong positive connotations in men compared with women, because it was equated with wisdom and high societal status.^8^ A famous example to illustrate this is Bellini's 65-year-old “Doge Loredan” (Figure 1A). Although the painting bears all the hallmarks of aging, including wrinkles, sagging skin, and hollow eye sockets, it does not convey frailty, weakness, or vulnerability. On the contrary, he poses gracefully, upright with a powerful gaze. Glorifying old age in men was a pillar upholding Venetian patriarchal gerontocracy and would not be challenged through the means of art. Artists would use female portraitures to depict the affliction of old age. An example is Giorgione's La Vecchia, who is also illustrated with visible signs of aging including wrinkles. However, unlike Doge Loredan, La Vecchia is painted with a hunched posture, crooked teeth, and a sorrowful gaze. In comparing these paintings, it becomes evident that portraiture functions as a lens through which cultural values are reflected. Men were esteemed for enduring intelligence, whereas women were prized for ephemeral beauty and fertility, illustrating a binary view of aging where male attractiveness remains timeless, whereas youthful female beauty diminishes rapidly. This remains relevant over the ages, where we find greater body dissatisfaction in women vs men, which lends itself to a greater proportion of females seeking aesthetic procedures to alter their body image to conform to societal standards.^9-11^

Of the 20 portraits selected for analysis from each school, 15 consisted of male sitters and 5 were female sitters. This appears to be in line with the overarching theme of lower representation of older women in Renaissance art.^12^ As discussed, this was in part because of the patriarchal gerontocracy that prevailed during the Renaissance period, particularly notable in Venetian society.^13^ Depictions of aging women are less numerous, and even entirely absent, from some portrait traditions, such as in Renaissance Florence.^14^ Hence, the ratio of male-to-female portraits in our study is likely an accurate reflection of the actual distribution of male-to-female portraits in existence.

Although these observations on aging and female representation are speculative and not directly supported by our data, they highlight an intriguing intersection between historical art practices and modern societal trends. Exploring these connections can provide valuable context for understanding how cultural values, past and present, shape perceptions of beauty and aging.

With regard to periocular aging, the main findings from our study establish a significant difference in the illustration of dermatochalasis and lower eyelid bags between the 2 schools of art. Whilst the Northern European artworks scored higher in both, Italian paintings scored lower. Because dermatochalasis and lower eyelid bags are recognized as early indicators of aging, the less severe depiction of these features in Italian portraits strongly implies that Italian art schools tended to represent aging less prominently. This study seeks to provide an objective basis to the art historians’ predominant school of thought that Italian Renaissance art projected a more youthful and idealistic portrayal of their muses.^15^ This can be further extrapolated to the notion that aging was considered undesirable in Southern Italian works of art.

The significant results from our study were from upper eyelid dermatochalasis and lower-lid bags; the lower eyelid laxity and lower eyelid tear troughs were not identified as being significantly different between the 2 renaissance schools. This could be because of a cultural phenomenon to focus on the prominent skin sagging of upper eyelids and bulging of lower eyelids as easily identifiable signs and indicators of aging, whereas forgiving hollowing and laxity changes in the lower lids. This emphasis may also reflect modern trends consistent with The American Society for Aesthetic Plastic Surgery statistics 2023 which show blepharoplasty among the top 5 aesthetic surgeries in the United States along with a significant increase of 9.4% from 2022 to 2023.^16^ This could be suggestive of human tendencies to focus on periocular changes, whether it is the Renaissance or our contemporaneous era.

This study demonstrates a convergence of objective scientific grading and the art historian's view regarding the variations between Southern Italian and Northern European Renaissance art schools. Expanding upon this groundwork, we can advance the discussion to explore potential theories elucidating the distinctions between these groups. The differences in the depiction of aging during the Renaissance period between Southern and Northern European art stem from a combination of cultural, artistic, and socioeconomic factors. In the Southern Renaissance, molded by classical ideals and patronage of the elite, artists emphasized symbolic and idealized representations of aging, reflecting humanist values and allegorical themes. Italian artists employed techniques such as linear perspective and chiaroscuro to create harmonious and timeless depictions. In contrast, the Northern Renaissance, defined by a growing merchant class and demand for realism, prioritized individualized and detailed portrayals of aging, reflecting Christian morality and everyday life. Northern European artists, innovating with oil painting and printmaking, captured the nuances of aging with greater realism, catering to a broader audience beyond the elite patrons of Italy. The cultural manipulation of reality in the portrayal of these images is a phenomenon also observed in modern times, particularly in the context of conventional and social media–based alterations of images.

The nature of this study posed some limitations. The age-matched focus of the study led to the selection of only well-known figures from the European Renaissance era, who were predominantly Caucasian. Consequently, portraits of sitters from other ethnic backgrounds were inadvertently excluded, resulting in selection bias. Observation bias is another limitation of this study. Although we attempted to mitigate it by employing 2 independent observers with aforementioned inter-rater reliability, the inherent subjectivity of grading may still influence the results.

Beyond its historical relevance, this work informs contemporary aesthetic practices by highlighting how symbolic representations influence perceptions of aging. Recognizing symbolic associations of features informs discussions with patients pertaining to aesthetic objectives and fosters considerations of psychological impact; balancing realism and idealism and contributes to overall aesthetic outcomes. It promotes responsible and patient-centered practices. Collaborating with art historians enhances interdisciplinary understanding, fostering a holistic approach to aesthetics and ultimately improving the quality of care and outcomes in aesthetic procedures. Finally, we aspire for this research to establish an objective framework, particularly for European artworks, that could assist in estimating the ages of subjects depicted in portraits, aiding in sitter identification and art historical dating.

## CONCLUSIONS

This study bridges the gap between subjective art interpretation and objective scientific analysis, offering new insights into how aging was portrayed in Renaissance portraiture. By applying modern grading scales to periocular features, the study suggests that Southern Italian artists idealized youth more strongly than their Northern European counterparts, who favored realism. These artistic choices could be considered a reflection on broader cultural attitudes toward aging, gender, and beauty. Hence, anatomic realism and cultural idealization within art do offer enduring lessons about aesthetic subjectivity and interpretation of beauty.

## Supplementary Material

ojag181_Supplementary_Data
