# Supplementary material for: The Portrayal of Periocular Aging Changes in Renaissance Art: A Case–Control Study to Compare Northern vs Southern Schools of Art
Source: Aesthet Surg J Open Forum. 2026 Sep 18;8:ojag181. doi: 10.1093/asjof/ojag181 (PMC13674795; doi:10.1093/asjof/ojag181)
Supplement: Supplementary file 1 [file ojag181_supplementary_data.docx]

Figure 1: Classification systems used for Upper and Lower eyelid Ageing changes:

Figure 1A JEC (Jacobs Eyelids Classification) for central dermatochalasis: Grading of central dermatochalasis based on lax skin level in comparison to eyelashes:

A, Normal (the upper eyelid skin is not touching the eyelashes).

B, Mild (the upper eyelid skin is touching the eyelashes).

C, Moderate (the upper eyelid skin is hanging over the eyelashes).

D, Severe (the upper eyelid skin is hanging over the eye).

Figure 1B LDC (Lateral Dermatochalasis Classification) for lateral dermatochalasis: Grading based on the relation of lateral lax lid to imaginary lines, one passing through lacrimal caruncle and the lateral edge of the upper eyelid and other passing through lower edge of the iris at the pupillary midpoint

Grade 0—absence of dermatochalasis, in the lateral region of the orbit.

Grade 1—lower edge of dermatochalasis (LED) is located above the intersection of the lacrimal caruncle with the edge of the upper eyelid.

Grade 2—between the intersection of the lacrimal caruncle with the edge of the upper eyelid and the lower edge of the iris at the pupillary midpoint, even when the LED is at the same level of the intersection of the lacrimal caruncle with the edge of the upper eyelid.

Grade 3—LED below the lower edge of the iris, even when it is at the same level of it.

Figure 1C ABL (Atrophy, Bulging, Laxity) classification for lower eyelid ageing changes

Atrophy: A0  No tear trough, No PMG (palpebromalar groove), No AMC (anteromedial cheek) volume loss

A1  Tear trough medial portion only

A2  Tear trough medial portion and PMG

A3  Tear trough medial portion and PMG and AMC volume loss

Bulging: B0 No bulging in the lower-eyelid-AMC area

B1 Lower eyelid bags/bulging only

B2 Malar festoons only

B3 Lower eyelid bags/bulging and Malar festoons

Laxity: L0  No lower eyelid laxity, No cheek laxity

L1  Lower eyelid laxity only

L2  Cheek laxity only

L3  Lower eyelid laxity and Cheek laxity

Figure 2: Comparison of Central Dermatochalasis scores using JEC classification between Italian and Northern Renaissance Schools of Art

Figure 3: Comparison of Lateral Dermatochalasis scores using LDC classification between Italian and Northern Renaissance Schools of Art

Figure 4: Comparison of Lower eyelid Bulging scores using ABL classification between Italian and Northern Renaissance Schools of Art
